# 

**Published:** 1879-10

**Authors:** 


### NEW BOOKS.

We have received the following books and periodicals:

Statistics, Medical and Anthropological, of the Provost-Marshal General's Bureau. 2 Vols. Quarto. Compiled by J. H. Baxter, A. M., M. D., by order of the Sec. of War.

This is an exhaustive treatise taken from the records of examination of over one million men, recruited for the late war.

---

National Board of Health Bulletin. No. 12. The organ of the National Board of Health, giving the sanitary condition of the cities and reports of existing epidemics.

---

GOOD COMPANY.—A literary magazine, well printed, with tasty cover and contents and no editroial head visible. GOOD COMPANY, Springfield, Mass.

---

ST. NICHOLAS.—Scribners' Illustrated Magazine for Boys and Girls. $3 a year. Scribner & Co., Publishers, New York.

The jolliest and very best magazine in this world, for the young folks. Nothing approaches it in the beauty of its stories and the elegance of illustration.

### SUBSCRIPTIONS RECEIVED

To Oct. 1, 1879.

In this column we will credit every subscription received during the last quarter. Subscribers will please notify us immediately if their payments are not duly credited. The State only is given—this, with the full name of the party subscribing, will be sufficient to indicate who is meant.

NEW YORK—Dr W D Saxton, John Brown, S C Burdick, F A Frasier, Dr C Allen, J W Henocksburg, Dr Jno Griffin, Dr P R Snodgrass, Dr Hiram Friedling, Dr Ansou Jourdan, Dr J T Covell, Dr L Armstrong, Dr J R Gumberling, Mrs S Cummings, Dr Anderson, Dr S M Hardt, Mrs L B Fuller, Mrs Jane Comstock, Sam'l Kline, Dr L Brown, Mrs Sam'l LaTrobe, Dr L D Clark, Dr Vanover, John Wintermute, Mrs Hal DeWitt, Dr O V Smith, Dr N P Banks, Dr U M Howe, Dr T T Simonson, Mrs J D Taber, Dr Ingersoll, N J Sanders, T B Crane, Sol Hersey, Dr Myron Clark.

PENNSYLVANIA—M G Shoemaker, Dr R S Pratt, W P Scharf, C S Sellard, Dr Jno Y Shindel, Dr D Blizzard, Dr Hollenstien, Dr B Bowns, Dr H Kong, Dr W Williams, Dr D Haywood, Dr S R Barnes, Dr E Gampert, Dr O Young, Dr Blakiston, Dr B Payne, Dr H Stevens, Dr D Donnelly, Dr John Walker, Dr Hiram Pennybaker, Mrs D Johnson, D N Bangs, John Gunberling, Sarah Billings, Dr P Snow.

ALABAMA—W R Chievhur, Dr D Chisholm.

DIST. COL.—F E Heiiberger.

ILLINOIS—Mrs L G Holley, Dr J D Groom, Dr L B Flemming.

MISSOURI—Ben H Wilson, Dr J I Levis, Dr J D Hoges.

MARYLAND—Louisa M Herbert.

NEW JERSEY—Sam'l E Burr, Dr B J Tinkham, Dr S I Williams, Dr Sam'l Ferguson, Dr H H Kalisle.

TENNESSEE—Isaac B Walton, John R Warkins, Dr C B B Frothingham.

WEST VIRGINIA—Dr I Titthouse, Dr O Lourey.

WISCONSIN—Dr James Johnson.

NO NAME—Louisville, Ky., $1.00, Harrisburg, Pa., $1.00

NO PLACE—John D Price, $2.00.

